# Photodegradation of Anti-Inflammatory Drugs: Stability Tests and Lipid Nanocarriers for Their Photoprotection

**DOI:** 10.3390/molecules26195989

**Published:** 2021-10-02

**Authors:** Giuseppina Ioele, Fedora Grande, Michele De Luca, Maria Antonietta Occhiuzzi, Antonio Garofalo, Gaetano Ragno

**Affiliations:** Department of Pharmacy, Health and Nutritional Sciences, University of Calabria, 87036 Rende, Italy; fedora.grande@unical.it (F.G.); michele.deluca@unical.it (M.D.L.); mariaantonietta.occhiuzzi@unical.it (M.A.O.); antonio.garofalo@unical.it (A.G.); gaetano.ragno@unical.it (G.R.)

**Keywords:** photodegradation, ICH Quality Guidelines, liposomes, niosomes, solid lipid nanoparticles

## Abstract

The present paper provides an updated overview of the methodologies applied in photodegradation studies of non-steroidal anti-inflammatory drugs. Photostability tests, performed according to international standards, have clearly demonstrated the photolability of many drugs belonging to this class, observed during the preparation of commercial forms, administration or when dispersed in the environment. The photodegradation profile of these drugs is usually monitored by spectrophotometric or chromatographic techniques and in many studies the analytical data are processed by chemometric procedures. The application of multivariate analysis in the resolution of often-complex data sets makes it possible to estimate the pure spectra of the species involved in the degradation process and their concentration profiles. Given the wide use of these drugs, several pharmaceutical formulations have been investigated to improve their photostability in solution or gel, as well as the pharmacokinetic profile. The use of lipid nanocarriers as liposomes, niosomes or solid lipid nanoparticles has demonstrated to both minimize photodegradation and improve the controlled release of the entrapped drugs.

## 1. Introduction

Non-steroidal anti-inflammatory drugs (NSAIDs) represent a class of drugs usually applied in the treatment of acute or chronic conditions involved in pain and inflammation [1]. These analgesic properties have been linked to their inhibitory activity of cyclooxygenases (COX), presents in two major forms, which convert arachidonic acid into various prostaglandins [2]. Several well-known compounds, such as Ketoprofen, Indomethacin, or Diclofenac, are active on the constitutive form COX-1, which plays an important role in cellular homeostasis. Other latest-generation compounds, such as Celecoxib, preferentially inhibit the form COX-2 and are highly effective as anti-inflammatory drugs, avoiding the adverse gastrointestinal side effects of NSAIDs. However, other studies suggest that prolonged use of COX-2 inhibitors may increase the incidence of cardiovascular side effects [3]. Recently, the use of NSAIDs has been safely used to relieve symptoms in patients with suspicion of Covid-19, and has not been associated with mortality or ventilator care in these patients [4]. Other pharmacological activities of NSAIDs are well recognized, such as the inhibitory effect on carcinogenesis and cancer spreading due to different mechanisms, including the induction of cell proliferation, apoptosis inhibition, and host’s immune response suppression [5].

NSAIDs can be classified according to their chemical structure or mechanism of action in: salicylates such as Acetylsalicylic acid; propionic acid derivatives such as Ibuprofen, Naproxen, Ketoprofen and Flurbiprofen; acetic acid derivatives such as Indomethacin, Diclofenac and Nabumetone; enolic acid (oxicam) derivatives such as Piroxicam; anthranilic acid derivatives (phenamates) such as Mefenamic acid; and selective COX-2 inhibitors (coxibs) such as Celecoxib [6].

The light sensitivity of these drugs has been studied mainly in commercial formulations [7,8] and in environmental matrices [9,10,11]. For example, the sunlight exposure of Diclofenac has been investigated in solution or topical formulations [7], but its photoproducts are frequently detected in seawater and other aquatic environments [9]. Likewise, Celecoxib has been detected in river water, in which it is not biologically degraded but only minimally altered after exposure to sunlight or high temperature [12].

Unfortunately, despite most of the anti-inflammatory drugs for topical use showing simplicity of application and minimal systemic absorption, adverse cutaneous photosensitivity reactions have been reported [13]. Such photosensitivity reactions are commonly due to an abnormal light-induced chemical reaction in which the drugs can form various photoproducts by absorbing radiation. This can cause oxidation of lipids, proteins, and deoxyribonucleic acid (DNA) and cause phototoxicity with errors during cell replication and consequently processes of mutagenesis, carcinogenesis, and cell death [14].

The sunlight-guided photocatalytic degradation of harmful drugs and chemicals can be exploited to eliminate them from the environment [15] or it can be reduced when it shows signs of danger to the environment or to humans. Several systems have been proposed to reduce light degradation [16,17,18]. The first approach in light protection consists of the use of suitable packaging materials that shield visible and/or UV sun radiation. Otherwise, the addition of light-absorbent excipients in the formulation is often proposed to provide the photoprotection of the drugs [7,19,20].

However, the incorporation of drugs into drug delivery systems (DDS) is now the most successful approach, and is able to reduce light degradation while simultaneously performing controlled release of drugs. Traditional DDSs have shown significant deficiencies, as they distribute agents non-specifically in the body, thus affecting both targets and healthy body cells. In contrast, nanoscale DDSs herald innovative nanotechnology in a wide range of potential therapies, including cancer [21]. Among the most studied incorporation systems of NSAIDs, vesicular matrices (i.e., liposomes and niosomes) and solid lipid nanoparticles have shown the best results [22].

Lipid nanosystems offer several advantages, such as the possibility of improving the stability of the incorporated compounds and allowing their modulated release action. For example, for drugs, it is possible to improve their solubility in water, their thermal stability, and increase their oral bioavailability, as well as protecting them during the digestive process (after oral administration). These systems can also improve their organoleptic and functional properties [23,24]. Recently, lipid-based nanocarriers were applied for the encapsulation of essential oils used as “natural food additives” in the preservation process of food such as cooked rice or rice flour in food industries [25].

This review provides an exhaustive collection of the most widely used approaches in the investigation of the photodegradation profile of NSAIDs and the lipid delivery devices proposed for their photoprotection.

## 2. Photodegradation Studies of NSAIDs

The quality of a drug and of the formulations containing it must be controlled and ensured during all phases of development. In this quality assurance system, the analytical methods adopted must be able to reliably measure, in addition to the active compounds, any degradation products present in the formulation.

### 2.1. ICH Quality Guidelines

The provisions relating to the methods of determining and applying the stability studies are provided in the ICH (International Conference on Harmonization) Guideline to guarantee the safety, efficacy and quality of the tested drugs. These rules describe the stability tests for the drugs over time in different environmental storage conditions (pH, temperature, light, air, and humidity) [26]. Among these, the ICH Q1A-R2 test defines the evaluation of temperature (in 10 °C increments), humidity (e.g., 75% RH or greater), oxidation, photolysis and hydrolysis over a wide range of pH values in solution or suspension. The ICH Q1B test includes photostability tests on both pure drug and its pharmaceutical forms, to verify the light sensitivity even of commercial products in marketing packages.

The photostability test can be performed by using two different light sources. According to option 1, an artificial daylight fluorescent lamp, combining visible and UV outputs produces an output similar to the D65/ID65 emission standard. D65 is the internationally recognized standard for outdoor daylight while ID65 is the equivalent indoor indirect daylight standard. For a light source emitting significant radiation below 320 nm, an appropriate filter may be fitted to eliminate such radiation. Option 2 is performed by combining a cool white fluorescent with a near ultraviolet lamp (320–400 nm), producing a spectral distribution from 320 nm to 400 nm with a maximum energy emission between 350 nm and 370 nm; a significant proportion of UV should be in the two bands 320 to 360 nm and 360 to 400 nm. An appropriate control of the temperature is required in all tests. dedicated instruments are usually equipped with an electronic device for controlling irradiation and temperature inside the box. Samples are generally irradiated in a λ range between 300 and 800 nm, by means of selected filters, producing an irradiation power from 250 to 765 W/m^2^. The cooling system keeps the temperature constant at 25 °C for radiant power values higher than 350 W/m^2^, while lower radiant power values do not induce an increase in the temperature inside the irradiation chamber. UV radiation can interact directly with the drug, causing molecular vibrations that could induce the breaking of bonds with the formation of free radicals or active species of oxygen, superoxide anions, hydroxyl radicals or single oxygen and consequently with the formation of photodegradation products [20].

The choice of the parameters in photodegradation experiments, including the type of radiation and exposure times, are discretionary but should always be justified. To assess the photodegradation profile of a drug, mild exposure conditions are used, applying less intense radiation and terminating studies in case of extensive decomposition. In forced degradation tests, on the other hand, the overall photosensitivity of a drug and the characterization of any degradation products are evaluated. In these studies, the samples must be placed in chemically inert and transparent containers and different exposure conditions can be used, depending on the photosensitivity of the substance involved and the intensity of the light sources used [26].

### 2.2. Analytical Techniques for Drug Determination

After optimizing a procedure for the drug recovery from the pharmaceutical or environmental matrix, it is necessary to establish the analytical method to measure the amount of the residual drug and its by-products. Considering the characteristics of the analytes, chromatographic procedures currently represent the most used technique, both in separation and quantization of the analytes.

In recent years, various chemometric methods, such as Multivariate Curve Resolution (MCR), have been applied to the data from spectrophotometric or chromatographic methods. These procedures have made it possible to simultaneously process the data recorded in multiple experiments and under different experimental conditions, making it possible to estimate the number of components involved in the full reaction process and the kinetic photodegradation profile of each component. In particular, the MCR methods make it possible to decompose an experimental data matrix from a chemical process into the pure contributions of the single components. The experimental data matrix, D, is decomposed into the product of two smaller factor matrices, C and ST:

D = C ST + E

where D is the data matrix obtained from the experimental spectral measurements and contains as many rows as absorption spectra recorded along the chemical process (time, reaction conditions, etc.), C is the concentration matrix of *n* components involved in the process, ST is the spectral matrix of the pure components and E contains the unexplained data variance [7,16]. In a photodegradation study, the number of species involved is difficult to determine, and chemical rank analysis can give a lower number of components than the real number of absorbing species, thus giving a rank deficiency. These problems could be removed by the simultaneous analysis of multiple experiments, under different conditions. When a drug is studied in the presence of lipid nanoparticles, MCR is able to elaborate the spectral data of the matrix components in the modeling step.

### 2.3. Application of the Photodegradation Test

Many NSAIDs are known to be sensitive to ultraviolet radiations (UVA or UVB) or visible light. The photodegradation of Acetylsalicylic Acid has been investigated in different conditions, in the absence or presence of excipients, in the presence of phosphate buffer (pH equal to 6.4, 7, and 8) or by interaction with NaOH 0.3 M [27]. The intermediate compounds formed by the process of photocatalytic oxidation or by other mechanisms of degradation, such as hydrolysis, electrophilic addition, electrons transfer, decarboxylation reaction, aromatic ring opening, and radical reaction, have been shown to be more toxic than the pure compound [28,29]. When Paracetamol was exposed to UV irradiation at 254 nm up to 96 h, it degraded by 50% after 24 h, producing a toxic photoproduct, identified as 1-(2-amino-5-hydroxyphenyl)ethanone. Identification and isolation of this photoproduct was carried out by HPLC and ESI/TOF/MS/MS. The luminescent bacteria test indicated that the photoproduct was more toxic than Paracetamol [30]. The photodegradation study of Ibuprofen was performed by GC-MS. The drug samples in solution were irradiated at 254 nm at a constant temperature of 20 °C for 60 min. The evaluation of the toxicity by *Vibrio fischeri* of the photolysis products indicated that this drug generated several photoproducts more toxic than the base compound [31]. The kinetics of Naproxen photodegradation was studied in aqueous solutions at 254 nm under de-aerated and aerated conditions, at pH 7.0 and constant temperature of 25 °C. The formation of two photoproducts, named 1-(6-methoxy-2-naphthyl)ethanol and 2-acetyl-6-methoxy-naphthalene, was evaluated by HPLC-DAD analysis [32]. Photostability of Diclofenac was investigated in liquid [33] and gel formulation, showing a clear degradation with the formation of three photoproducts presenting a quinone imine structure, probably obtained by decarboxylation and oxidation followed by dehalogenation and cyclization of the drug. In gel formulation, the degradation process has been monitored by applying the MCR technique to the UV spectral data from samples exposed to stressing irradiation. In this case, the photodegradation rate of Diclofenac in gel was very fast, with a residual content of 90% only after 3.90 min under a radiant exposure of 450 W/m^2^ at a temperature of 25 °C [7,16].

The stability of Celecoxib was studied by HPLC-DAD by exposing the methanol solutions, prepared in the range of 0.1–2 μg/mL, to various conditions of forced degradation in the presence of acidic and basic solutions or by exposure to light and heat. The photochemical profile was verified by exposing the drug to direct sunlight for 30 min, demonstrating the stability of this compound under these degradation conditions while the effect of the temperature has been studied by heating the acidic mixture for 30 min at 80 °C, and basic mixture for 10 min at 80 °C [29].

Table 1 summarizes the analytical techniques used in the most widely applied stability indication methods.

## 3. Characteristics of Lipid Nanocarriers

The number of studies on nanocarriers in the pharmaceutical field is constantly growing in terms of disease diagnosis and treatment. Indeed, new formulations of drugs entrapped in nanocarriers can lead to an improvement in the pharmacokinetic profile of the drugs and their protection from chemical or physical degradation [37,38].

Various types of nanocarriers are available, based on the different chemical characteristics of the polymers or lipids used for their preparation. Thus, nanocarriers can be classified as organic-based (as polymeric frameworks, lipid-based frameworks, liposomes and nanoemulsions), inorganic-based (as metallic nanostructures, silica nanoparticles and quantum dots), or hybrid combinations of both [37]. The simplest liposomal systems are composed of natural phospholipids, such as lecithin, which are also the main components of the biological membranes. These phospholipids can spontaneously self-assemble in aqueous medium forming one or more compartments in which the drugs are incorporated. They can act as carriers of hydrophilic (in the aqueous compartments) or lipophilic molecules (inside the lipid bilayers) [39].

Several lipid nanocarriers are used for the skin delivery of NSAIDs. In the management of rheumatoid arthritis, transdermal drug delivery has attracted increasing attention with respect to the parental route in order to overcome the limitations of systemic side effects due to the continuous use of corticosteroids [40]. Different nanocarriers have been applied to enhance the permeation of NSAIDs through the layers of the skin and reach the site of inflammation, such as liposomes and niosomes.

### 3.1. NSAIDs in Liposomes

Indomethacin loaded in liposomes exhibited more sustained in vivo anti-inflammatory effect due to the formation of a drug reservoir in the stratum corneum layer [41]. Due to the high encapsulation efficiency, these systems guarantee both a sustained release of the drug and a reduction of the drug content, thus decreasing the potential of unwanted off-target effects. These matrices are non toxic and allow for targeted administration to inflamed tissues, as demonstrated for example by the liposomal matrix incorporating Celecoxib [42].

### 3.2. NSAIDs in Niosomes

Liposomes have some limitations due to the high cost of formulation, lack of stability at various pH, and limited shelf-life due to the rancidity of the lipids [40]. In recent years, therefore, new vesicular systems have been studied in which the phospholipid content of liposomes has been replaced with non-ionic surfactants and cholesterol. Such vesicles, named niosomes, show better chemical stability, longer shelf-life, and lower cost thanks to the use of inexpensive non-ionic surfactants. Furthermore, studies carried out on the topical treatment of rheumatoid arthritis have shown a better skin penetration of drugs. For example, Etodolac and Etoricoxib [43] have been incorporated into niosomes in gel formulation, while Piroxicam in niosomes [44] has been incorporated into a transdermal patch.

### 3.3. NSAIDs in Solid Lipid Nanoparticles

**Figure 1:**
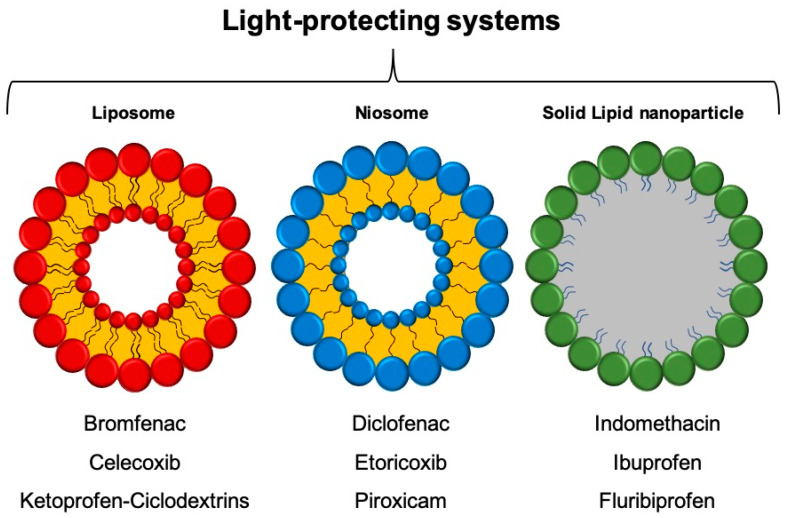
Schematic representation of liposome, niosome and SLN systems and known examples of entrapped NSAID drugs.

More advanced lipid-based carrier systems can potentially improve the bioavailability of highly hydrophobic, poorly water-soluble and/or lipophilic drugs. In fact, solid lipid nanoparticles (SLN) (ranging between 100 and 1000 nm) are colloidal lipid carriers that contain solid lipids, dispersed in water or in an aqueous surfactant solution. These lipids are prepared by using fatty acids, monoglycerides, diglycerides, triglycerides, waxes and steroids, and, depending on the preparation method, they can be used for both hydrophilic and hydrophobic drugs. In addition, to modulate drug release, such systems can protect the drugs from chemical decomposition [39,45]. Considering the limited loading capacity of the drug due to the presence of solid lipids, other SLN systems have been developed containing liquid lipids in a solid lipid matrix stabilized with biocompatible emulsifiers, named nanostructured lipid carriers (NLC). The NLC matrices, easily produced and entirely devoid of any organic solvent, are capable of encapsulating large amount of drug, have good long-term storage stability and can be used for oral administration of poorly water-soluble and low-bioavailability drugs [45]. Figure 1 shows the scheme of the most used lipid nanocarriers and examples of entrapped NSAIDs.

Flurbiprofen [46], Ibuprofen [47] and Piroxicam [48] have been loaded in SLNs in topical gel with high encapsulation efficiency, demonstrating that the concentration of lipid and surfactant plays an important role in the entrapment of the drugs. In the treatment of ulcerative colitis, a novel biocompatible nanoformulation for Celecoxib has been developed with colon specific characteristic. This NLC formulation has shown favorable characteristics: sustained release of the drug in physiological buffer solution, cytocompatibility for the normal cells, non-toxicity, safe for enteral human use, and cost-effectiveness [45].

## 4. Lipid Nanocarriers for Photoprotection of NSAIDs

Drugs are exposed to natural or artificial light throughout their pharmaceutical life, from their manufacture until dispensation or even after administration. Liquid preparations are usually less stable than solid formulations for the same drug substances. Several approaches have been proposed to protect NSAID compounds from light. To realize light-stable formulations, the use of light-absorbing agents represents one of the most widely investigated applications. The photoprotective effect of different ultraviolet (UV) filters has been evaluated with promising results in topical formulations of Ketoprofen, by adding butyl methoxy dibenzoylmethane [19]. The photostability of Diclofenac has been greatly increased by adding light absorbers such as octisilate, octyl methoxycinnamate and a combination of them to the gel formulations [7]. The inhibitory effect of Ascorbic Acid on Paracetamol, Ibuprofen, and Ketoprofen exposed to UV-B radiation has been studied with satisfactory results [20]. A clear improvement in the light protection of NSAIDs in topical formulations has been shown by entrapping the drugs into supramolecular matrices as cyclodextrins [49,50]. Photoprotection of Diclofenac, Ibuprofen and Naproxen have been approached through the incorporation of the drugs in several cyclodextrins [7]. Four photodegradation products were identified after light exposure of Piroxicam in methanol solution. The inclusion of this drug in 2-hydroxypropyl-cyclodextrin successfully increased the drug photostability by offering protection from daylight for up to 30 days [36].

Other NSAIDs have been combined in micro- or nanoemulsions to increase light-stability. Nabumetone and a newly synthesized analog (7-methoxy-2,3-dihydro-*1H*-cyclopenta[*b*]naphthalene-1-one) have been formulated in microemulsion for topical use to achieve better photostability and pharmacokinetic profile. Stability tests on both the compounds have shown a significant increase of photostability in liquid microemulsion and microemulsion-in-gel, compared to ethanol solution and plain gel. In addition, permeation experiments on the microemulsion-in-gel formulations have shown a better performance compared to the plain gel for both the compounds, highlighting the potential of the microemulsions as delayed drug delivery systems [35]. In developing therapeutic alternatives for the management of pain and inflammation, Ferreira et al. defined a combination of pomegranate seed oil and Ketoprofen in nanoemulsions. These nanoemulsions were demonstrated to be a stable system, presenting advantages over conventional emulsions due to the smaller droplet size. This also makes it possible to overcome the main limitation to the long-term therapeutic application of Ketoprofen linked to the harmful effects on the gastrointestinal tract. This formulation was able to both promote controlled drug release and drug protection against chemical, enzymatic degradation and photodegradation [51]. Table 2 summarizes all the proposed formulations used in protecting NSAIDs from light.

In the last decade, all the lipid inclusion matrices have also been studied as systems for preventing the degradation of NSAIDs [24]. These lipid carriers, SLNs in particular, have gained more importance because of their uniform size, smaller surface area, and high drug-loading capacity. The stability of a drug has been proved to proportionally increase with its incorporated quantity and, moreover, the lipid nanoparticles have the ability to scatter and reflect UV radiation. For these reasons, the application of lipid nanoparticles in drug formulations could improve the therapeutic efficacy by maintaining a controlled drug delivery and protecting the drug from degradation.

### 4.1. Photoprotection of NSAIDs in Liposomes

NSAIDs can form reversible interactions, such as ionic and hydrophobic bonds, with phospholipids such as phosphatidylcholine [57]. For example, the interactions between liposomes modified with chitosan, a mucoadhesive cationic polymer, have been studied to favor the application of Bromfenac on the retina [53]. This formulation has been prepared using the calcium acetate gradient method in which the negatively charged lipid dicetylphosphate has been incorporated into liposomes, forming an anion layer and preventing coalescence. Drug entrapment efficiency was greater than 90% using this method when the optimal concentration of chitosan has been selected at 0.15%. A formulation of Celecoxib entrapped inside the small, lamellar PEGylated liposomes composed of Leciva S90, cholesterol and methoxy polyethylene glycol di-stearoyl ethanolamine was prepared by Dave V. et al. through a thin-film hydration method using different molar ratios of drug to lipid. These liposomes were spherically shaped, with a smoothen surface in the periphery. The encapsulation efficiency depended on various parameters like the ratio of lipids and the molar ratio of drug to total lipids. A stability study of the optimized formulation was performed according to the ICH rules demonstrating the suitable storage condition at 4 °C. Thus, the use of this entrapping system can offer both efficient release of Celecoxib and easy administration via the parenteral route, by overcoming the problems caused by the free drug [56].

Liposomal matrices easily incorporate hydrophilic drugs into the aqueous core and then release them gently with minimal effect on the stability of the liposome. In contrast, the incorporation of lipophilic drugs into the lipid bilayer can interfere with the stability of liposomes [58]. Therefore, drug–cyclodextrin complexes have been developed in recent years that can make the drug soluble in water which are then loaded into liposomes [59]. This combined approach of cyclodextrin complexation and entrapment in liposomes has been investigated to develop a topical formulation of Ketoprofen [52]. This drug has been complexed with β-cyclodextrin and hydroxypropyl-βcyclodextrin using co-evaporation and sealed-heating methods. Liposomes consisting of phosphatidylcholine and cholesterol (60%/40%, *w*/*w*) have been prepared with different techniques, such as thin layer evaporation, freezing and thawing, extrusion through microporous membrane, and reverse phase evaporation method, obtaining, respectively, multi-lamellar vesicles, frozen and thawed multi-lamellar vesicles, small uni-lamellar vesicles and large uni-lamellar vesicles. The prepared complexes have been characterized by differential scanning calorimetry, demonstrating the influence of the cyclodextrin complex on the size of the liposomes and not on their lamellar structure.

### 4.2. Photoprotection of NSAIDs in Niosomes

The new generation of vesicular nanocarriers is represented by niosomes, self-assembled vesicles composed of non-ionic surfactants with/without adequate amounts of cholesterol or other amphiphilic molecules. Niosomes have a mono- or multilamellar structure like liposomes, being able to incorporate both lipophilic and hydrophilic bioactive substances. However, they have lower production costs and greater stability during storage [60]. Photostability studies have been performed on topical formulations containing Diclofenac in niosomal gels and compared to the commercial formulations containing the drug and standard gels prepared according to the Pharmacopoeia. Niosomal vesicles have been prepared with Span 60 or Tween 60 as multilamellar systems by adopting lipidic film method. The photodegradation profiles have been monitored by MCR applied to the spectral data, allowing to estimate spectra and concentration profiles of parent compound and by-products. Characterization of niosomes and permeation experiments have also been carried out to verify the performance of the prepared formulations. Under a radiant exposure of 450 W/m^2^, light stability increased significantly when the drug was entrapped in niosomal systems in presence of 5% ascorbic acid. Furthermore, the permeation capability of Diclofenac has been found to be about three times higher than that measured on the commercial gel [16,54].

### 4.3. Photoprotection of NSAIDs in Solid Lipid Nanoparticles

Another alternative to liposomes is represented by SLN and NLC. The latter have shown greater solubility of the incorporated drugs than SLN. Photostability studies have demonstrated the efficiency of these vesicular systems in preserving drugs from light photodegradation [61,62]. For example, Indomethacin has been loaded in SLNs and NLCs to investigate their potential use in topical ocular delivery. Chitosan (0.1% *w*/*v*) has been incorporated into the aqueous phase prior to preparation of the SLNs for their surface modification. The proposed formulations increased drug loading capability, entrapment, and delivery to anterior and posterior segment ocular tissues. Chemical stability has been investigated in storage conditions, demonstrating that the variation of the lipid component in the colloidal framework can improve drug release characteristics and chemical stability [55].

## 5. Conclusions

Photodegradation of drugs represents a problem that has emerged in recent decades, and the definition of photoprotective systems is of fundamental importance in the modern pharmaceutical industry. Several strategies have been proposed to improve the tendency of some drugs to degrade when exposed to light. This review reports the main lipid nanocarrier matrices proposed for drug formulation and their application in ensuring a valid photoprotection of the incorporated drugs. Lipid nanocarriers are characterized by the trapping of the drug into a cavity of their structure, involving only weak binding interactions. They have several advantages as drug carriers, especially for topical administration, and have been shown to both improve the pharmacokinetic profile and significantly increase the light stability of the non-steroidal anti-inflammatory drugs.

## Figures and Tables

**Table 1:** Determination methods of anti-inflammatory drugs in pharmaceutical or environmental matrix.

Drug Class	Drug	Pharmaceutical Formulation	Determination Method	References
Salicylates	Aspirin	Aqueous solution	LC/MS	[28]
		Solid state	FTIR	[28]
		Aqueous solution	GC/MS	[27]
Propionic acid derivatives	Ibuprofen	Aqueous solution	GC-MS	[31]
	Naproxen	Aqueous solution	HPLC-DAD	[32]
	Ketoprofen	Aqueous solution	HPLC-DAD	[8]
		Aqueous solution	HPLC-MS	[34]
Acetic acid derivatives	Diclofenac	Gel formulation	UV-Vis/MCR	[7,16,33]
		Aqueous solution	HPLC/MS	[33]
	Nabumetone	Gel formulation	UV-Vis and MCR	[35]
Aniline derivatives	Paracetamol	Aqueous solution	HPLC 7/ESI/TOF/MS/MS	[30]
Enolic acid derivatives	Piroxicam	Methanol solution	FTIR	[36]
Selective COX-2 inhibitors	Etoricoxib	Aqueous solution	UV-Vis	[10]
	Celecoxib	Methanol solution	HPLC-DAD	[29]

**Table 2:** Photo-protective pharmaceutical formulations of anti-inflammatory drugs.

Drug	Pharmaceutical Formulation	Photo-Protective System	References
Ibuprofen	Aqueous solution	Ascorbic acid as UV absorber	[20]
	Aqueous solution	Methyl-β-cyclodextrins	[49,50]
Naproxen	Aqueous solution	Methyl-β-cyclodextrins	[49,50]
Ketoprofen	Gel	Methoxy dibenzoylmethane as UV absorber	[19]
	Aqueous solution	Ascorbic acid as UV absorber	[20]
	Emulsion	Nanoemulsion	[51]
	Aqueous solution	β-cyclodextrin and hydroxypropyl-βcyclodextrin in liposomes	[52]
Bromfenac	Aqueous solution	Liposomes with chitosan	[53]
Diclofenac	Gel	Octisilate and/or octyl methoxycinnamate as UV absorbers	[7]
	Aqueous solution	Methyl-β-cyclodextrins	[7,49,50]
	Gel	Niosomes	[16,54]
	Gel	Niosomes and 5% ascorbic acid	[16,54]
Nabumetone	Gel	Microemulsion	[35]
Paracetamol	Aqueous solution	Ascorbic acid as UV absorber	[20]
Piroxicam	Aqueous solution	2-hydroxypropyl-cyclodextrin	[36]
Indomethacin	Aqueous solution	SLNs	[55]
	Aqueous solution	NLCs	[55]
Celecoxib	Aqueous solution	PEGylated liposomes	[56]
